# Shopping Detail Information and Home Freezer Sampling Confirmed the Role of Commercial, Modified-Atmosphere Packaged Meatballs as a Vehicle for Listeriosis in Finland

**DOI:** 10.3389/fpubh.2019.00216

**Published:** 2019-08-06

**Authors:** Riikka Keto-Timonen, Annukka Markkula, Jani Halkilahti, Reetta Huttunen, Sirpa Räsänen, Saara Salmenlinna, Anne Heikkilä, Mia Puisto, Maria Närhinen, Marjaana Hakkinen, Hannu Korkeala, Katri Jalava

**Affiliations:** ^1^Department of Food Hygiene and Environmental Health, University of Helsinki, Helsinki, Finland; ^2^Microbiological Food Safety Unit, Finnish Food Authority, Helsinki, Finland; ^3^Health Security Department, National Institute for Health and Welfare, Helsinki, Finland; ^4^Department of Internal Medicine, Tampere University Hospital, Tampere, Finland; ^5^Infectious Diseases Control Unit, Tampere, Finland; ^6^Environmental Health Office of Etelä-Satakunta, Säkylä, Finland; ^7^Environmental Health Unit, Mikkeli, Finland; ^8^Microbiology Research Unit, Finnish Food Authority, Helsinki, Finland; ^9^Department of Mathematics and Statistics, University of Helsinki, Helsinki, Finland

**Keywords:** *Listeria monocytogenes*, foodborne, ready-to-eat foods, meatball, listeriosis, loyalty card, surveillance

## Abstract

In November 2016, an elderly patient was diagnosed with *Listeria monocytogenes* bacteremia in Finland. Grocery store loyalty card records and microbiological investigation of foods found in the home fridge and freezer of the patient revealed commercial, modified-atmosphere packaged meatballs as the source of the infection. Investigation of the meatball production plant revealed that the floor drain samples were contaminated with the same *L. monocytogenes* strain as those isolated from the patient and meatballs. Ready-to-eat meatballs were likely contaminated after heat treatment from the production environment before packaging. Long-term cold storage, modified-atmosphere conditions, and the absence of competing bacteria presumably enhanced the growth of *L. monocytogenes*. We recommend that collection of shopping details and home fridge and freezer sampling should be part of surveillance of all cases of *L. monocytogenes* infections to complement information obtained from in-depth interviews.

## Introduction

Listeriosis is a severe, mainly foodborne infectious disease (1). While large outbreaks of listeriosis are relatively rare, small clusters of a few cases are more often observed (2–4). Susceptible populations include the immunocompromised, newborns, pregnant women, and the elderly (5). An increasing trend in human listeriosis has been observed in the EU since 2008 and the majority of cases are reported in persons >64 years old (6). This is probably due to increases in both the aging and immunocompromised populations and the increased use of risk foods such as ready-to-eat (RTE) foods with modified-atmosphere packaging and extended durability (6–8). The course of illness is often severe; most patients develop sepsis or meningitis and the mortality is approximately 30% (9). Finland is one of the high-risk countries for listeriosis (6). Identifying the source of the infection is challenging since the incubation period may range from 1 to 67 days (10). Therefore, the vehicle for infection often remains unidentified in sporadic cases (11).

*Listeria monocytogenes* is a facultative anaerobic bacterium. Various food vehicles have been linked to listeriosis outbreaks. Traditionally, deli meats, hot dogs, jellied meats, other processed meat products, soft cheeses, and unpasteurized dairy products have served as sources of infection (12). During the last decade, large outbreaks have also been caused by fresh produce such as sprouts, melons, stone fruits and apples (13–16). In Nordic countries, the most common vehicles for listeriosis include vacuum-packaged fish or RTE meat products (7, 17–23). However, commonly recognized high-risk foods are not always associated with sporadic listeriosis cases (11, 24). *L. monocytogenes* contamination of finished food products often arises from the processing equipment and environment (14, 25–29). Several studies have shown that *L. monocytogenes* may persist in food processing plants for prolonged periods of time (26, 30–33). Processing lines and machines with complex structure that hinder efficient cleaning are especially prone to persistent contamination (27, 30, 32, 34, 35).

In Finland, most listeriosis cases are sporadic (2) and more research is needed to identify possible food vehicles. An elderly patient infected with *L. monocytogenes* was reported in November 2016 from one municipality in Finland. The patient was interviewed to determine the source of the infection and to prevent further cases. Epidemiological and microbiological investigations revealed commercial meatballs as the source of infection. Although RTE meatballs have been recalled due to *L. monocytogenes* contamination (36), to our knowledge there have been no previous reports of clinical listeriosis caused by commercial meatballs.

## Materials and Methods

### Descriptive Information and Epidemiological Investigation

An over 90 year old male presented with oliguric acute renal failure and heart failure and was admitted to regional hospital in November 2016. The patient had chronic diseases including atrial fibrillation, cardiac insufficiency, and renal failure. He had signs and symptoms of deep venous thrombosis and leg swellings. The laboratory tests included three blood cultures, complete blood count, creatinine level, sodium, potassium, CRP, fecal culture, and liver enzymes. The patient had high white blood cells exceeding 30 (x10E9/l) and mildly decreased platelet count, 95 (x10E9/l) indicating the possibility of severe infection. His CRP level was normal on hospital admission (4 mg/l), but increased to 204 mg/l 4 days after hospital admission. During the first day of hospital treatment, cellulitis was suspected and the patient was started with intravenous cefuroxime. He was also suspected to have acute cardiac failure and was treated with diuretics. However, blood cultures revealed *L. monocytogenes* growth in two consecutive blood cultures. The patient had no signs of meningitis, sepsis, or haemodynamic instability. After the blood cultures revealed *L. monocytogenes*, bacteraemia was managed with ampicillin 2 g three times a day. After 2 days of treatment, the CRP value decreased and blood cultures were not taken after or during ampicillin treatment. The hospital immediately informed the local environmental health authorities about the listeriosis case and the local outbreak control team of food safety and health authorities was activated to prevent further cases. Condition of the patient improved and he was transferred to health centers department for further rehabilitation after 17 days of hospital treatment.

An in-depth interview was performed in the hospital to obtain information about the food preparation practices and diet of the case to identify possible high-risk foods for *L. monocytogenes*. The case lived on his own in an owner-occupied flat. He utilized a home help service to deliver food at home and a weekly RTE food service. He bought food from supermarket A and the home help service also delivered to him food from supermarket B. Permission to perform a home inspection of the fridge and freezer was obtained from the case patient and his close relatives. In addition, permission was sought to obtain loyalty card (grocery store loyalty card or credit card) records for a period of May to November 2016 (6 months prior to the onset of symptoms). Ethical approval was not required as this outbreak investigation was part of acute public health response as permitted by the Finnish health legislation.

### Inspection of Home Food Delivery Service

The municipal food safety authority inspected the kitchen facilities of the home food delivery service. During the inspection, details of the meals served during the regular 7 week rotation were screened to identify possible listeria risk foods and hygienic measures in the kitchen were evaluated.

### Microbial Analysis of Patient and Food Samples

Routine samples were taken from the patient in the regional hospital according to standard protocols after suspicion of infection was raised. The *L. monocytogenes* isolate was sent to the Expert Microbiology Unit at the National Institute for Health and Welfare (Helsinki, Finland) for further characterization by whole genome sequencing (WGS).

Food sampling from the domestic fridge and freezer of the case was targeted to common foods and foods that are known to be potential sources of *L. monocytogenes*. Altogether 21 food samples obtained from the kitchen of the patient were examined using the *Listeria* enrichment ISO 11290-1 (37) or enumeration ISO 11290-2 (38) procedure, or both. *In-situ* DNA isolation and pulsed-field gel electrophoresis (PFGE) of *L. monocytogenes* isolates were performed as described by Autio et al. (39) using *Asc*I (New England Biolabs, Ipswich, USA) and *Apa*I (Roche, Mannheim, Germany) restriction enzymes. The resulting PFGE patterns were analyzed using BioNumerics software v. 5.10 (Applied Maths, Sint-Martes-Latem, Belgium) and compared with patterns of *L. monocytogenes* isolates (*n* = 1,641) in the PFGE database of the Department of Food Hygiene and Environmental Health (DFHEH, University of Helsinki, Finland) originating from food and environmental samples. Environmental sampling was performed in the food production premises of the implicated producer (producer C) by local food safety authorities.

All food and environmental isolates of *L. monocytogenes* isolated during this investigation were subjected to WGS at the Institute of Molecular Medicine Finland (Helsinki, Finland). The Nextera XT DNA library preparation kit (Illumina Inc., San Diego, USA) was used for library preparation and sequencing was performed on the Illumina HiSeq system (Illumina). Sequence data were analyzed at the National Institute for Health and Welfare. This analysis included *de novo* assembly using Velvet assembler (version 1.1.04) (40), classical seven-gene multilocus sequence typing analysis (41), allele calling (42) utilizing a core genome (cgMLST) and accessory genome schema, and visualization of the results in a Minimum Spanning Tree. The cgMLST analysis schema contained 1,504 targets, defined by using one Finnish outbreak strain as reference genome (Genbank accession number NC_017547.1) and 45 *L. monocytogenes* complete genomes from Genbank. In order to increase discrimination, the shared accessory genome of the nine isolates studied, were also used. All previous steps in the workflow were included in the Ridom SeqSphere+ software (version 5.1.0) (43).

## Results

### Patient Isolate

**Figure 1:**
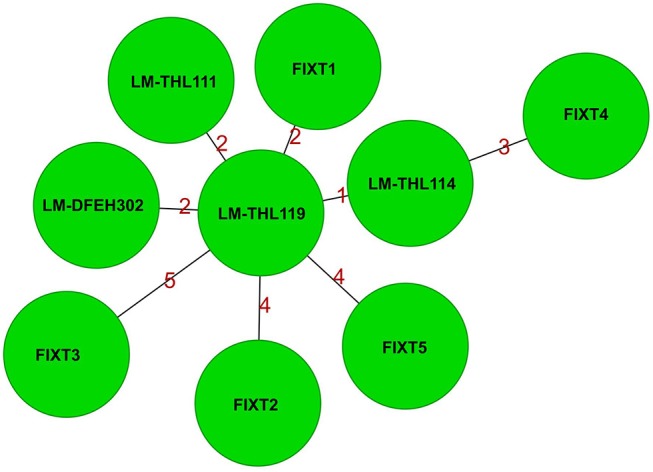
Minimum spanning tree based on 2,597 shared core genome and accessory genome targets of *Listeria monocytogenes* isolates. All isolates belong to seven-gene multilocus sequence type (ST) 124. All ST 124 isolates clustered together with less than seven allele differences (<0.3% difference). The cluster consisted of three isolates from three different patients (the index patient LM-THL119, LM-THL111 from year 2014, and LM-THL114 from year 2015), four food isolates from meatballs (LM-DFEH302, FIXT3, FIXT4, and FIXT5), and two environmental isolates from the meatball production plant (FIXT1 and FIXT2). Each node represents a single isolate and the values on the connecting lines illustrate the number of allele differences.

The patient *L. monocytogenes* isolate (LM-THL119) belonged to sequence type (ST) 124 (Figure 1). Two additional ST 124 isolates were recovered from patients in age groups of 30-40 and 70–80 years in Finland in 2014 (LM-THL111) and 2015 (LM-THL114), respectively. No loyalty card information or sampling of home fridges was performed for these additional listeriosis cases.

### Trace Back of Foods and *L. monocytogenes* Isolates Recovered From Food and the Food Production Environment

During the in-depth interview, the case reported having eaten vacuum-packed fish. The in-depth interview did not include meatballs. The hygienic measures in the kitchen of the home food delivery service were appropriate and the meal service menu did not include particular risk foods for listeria.

The fridge and freezer of the patient were quite full and there were some flaws in the tidiness. *L. monocytogenes* was isolated from two self-packed meatball bags obtained from the freezer compartment of the refrigerator of the case (isolates FIXT3, FIXT4, FIXT5, and LM-DFEH302); from the other food samples *L. monocytogenes* could not be isolated. Meatballs were purchased from supermarket B according to loyalty card information. The amount of *L. monocytogenes* in the meatballs was 170 and 190 cfu/g in the two different bags. To identify the producer of the self-packed *L. monocytogenes-*positive meatballs, the meatballs were visually compared to six commercial meatball products identified from the loyalty card shopping records of the patient. Different producers use various methods for preparation and different ingredients and cooking conditions. Thus, each studied meatball product had a unique and distinct appearance. Based on the comparison, both self-packed meatball bags could be traced back to producer C. The meatballs were purchased on 19 October with a use-by-date of 7 November. The meatballs of producer C were sold nationwide but not exported to other countries.

An inspection and environmental sampling were performed in the food production premises of producer C by local food safety authorities. *L. monocytogenes* was isolated from the floor drain of the high hygiene environment of the production plant (isolates FIXT1 and FIXT2). WGS and PFGE typing of *L. monocytogenes* isolates from patient, meatballs, and environment showed that the isolates shared an identical PFGE pattern and belonged to ST 124. The patient isolates and all food and environmental isolates shared 2,597 common target loci out of a possible 2,724 loci included in the core and accessory genome analysis schema. All the isolates clustered together with less than seven allele difference (i.e., difference <0.3%) (Figure 1).

Comparison of the *Asc*I and *Apa*I restriction profiles of *L. monocytogenes* isolated from meatballs with those in the PFGE database of DFHEH revealed 10 identical PFGE patterns that originated from four different food production plants. Four of the isolates with an identical PFGE profile originated from a food production plant that produced RTE foods. The isolates were recovered on four separate occasions between November 1998 and February 1999 from a conveyor in a compartment that produced RTE minced meat products. Three of the isolates with an identical PFGE profile originated from the production environment of another RTE food production plant and one of the isolates was recovered from raw sausage batter of a meat production plant. Two of the isolates with an identical PFGE pattern were from raw material (sweet pepper) and a finished food product (pizza) from a RTE food processing plant.

## Discussion

For the first time, commercial, modified-atmosphere packaged meatballs stored in the home freezer of a patient were identified as a vehicle for human listeriosis. The source was identified using loyalty card information and home refrigerator sampling. All patient, food and environmental isolates belonged to ST 124 and observed allele differences were minor (<0.3% of the shared targets) suggesting common origin of the isolates. RTE meatballs were likely contaminated from the production environment after heat treatment prior to packaging. Long-term cold storage, modified-atmosphere conditions, and the absence of competing bacteria presumably enhanced the growth of *L. monocytogenes*. In order to eliminate the listeria contamination, producer C carried out thorough cleaning and disinfection of equipment and production environment as well as made structural changes and maintenance operations to the production line and environment. The success of the elimination was ensured by enhanced sampling of both meatballs and the production environment.

An identical PFGE type has also been previously isolated from the food production environment, raw materials, and products of different food processing plants. In one RTE food processing plant, isolates with an identical PFGE profile were repeatedly found on a conveyor, suggesting that the isolate may persist in the food production environment. Although further genomic testing was not performed for these earlier isolates, it is noteworthy that these isolates with identical PFGE profile may have properties that aid them to adapt to environmental niches in the food processing environment.

Identification of the vehicle food in sporadic listeriosis cases is challenging (11). Previous studies have shown that utilization of supermarket loyalty card records and testing of food items obtained from the homes of the case patients are effective ways of identifying vehicles of large foodborne outbreaks (44–48). However, the present investigation showed that grocery store loyalty card records and sampling of the home refrigerator and freezer of the case can also aid in source identification of sporadic listeriosis cases. Due to the long incubation time, in-depth interviews may result in recall bias and inaccuracy of information; this is a special concern when interviewing older people whose memory may be impaired.

There was an abundance of foods in the freezer of the case, which is a common practice among elderly persons. It may be that elderly people favor long storage of foods and freeze them only close to the best-before date, when even initially low levels of *L. monocytogenes* may have turned into high numbers despite refrigeration. In addition, they may use cooking practices that are inadequate to destroy *L. monocytogenes* or no cooking after thawing.

*L. monocytogenes* bacteremia may have an atypical presentation in the elderly. In this case, highly elevated leucocyte count and decreased platelet count were indicative of infection, although the patient did not have classical signs and symptoms of *L. monocytogenes* bacteremia. Elderly people are highly susceptible to listeriosis (12) and the infectious dose can be very low. However, in the current case, the amount of *L. monocytogenes* in the meatballs was >100 cfu/g. This also underlines the importance of national surveillance and vigilance for similar types of cases. Meatballs are commonly eaten by Finnish people, also as cold snacks. As these types of meatballs are sold nationwide and may be consumed after freezing and thawing, the occurrence of cases may be sporadic in both time and place. As the meatballs were widely sold in high quantities and have previously not been labeled as high risk foods, we also checked that no excess number of listeriosis cases were observed during the same time period.

We recommend that collection of shopping details and home freezer sampling should be part of the surveillance of all cases of *L. monocytogenes* infections to complement information obtained from in-depth interviews. As both the proportion of listeriosis cases and fatal cases has increased in persons >64 years (and particularly in persons >84 years), it is also important to raise awareness of listeriosis and risk foods in elderly people (6).

## Data Availability

The datasets generated for this study can be found in European Nucleotide Archive, PRJEB31548 (https://www.ebi.ac.uk/ena/data/view/PRJEB31548).

## Author Contributions

AH, RH, SR, RK-T, and KJ carried out the epidemiological investigation. MP, MN, AM, and KJ conducted trace back of the meatballs. Patient and relative interviews were done by RH, SR, and KJ. RK-T, AM, JH, SS, MH, and HK did the microbiological and sequencing analysis of the listeria isolates. RK-T and KJ drafted the manuscript and all co-authors contributed to manuscript revision.

### Conflict of Interest Statement

The authors declare that the research was conducted in the absence of any commercial or financial relationships that could be construed as a potential conflict of interest.
